# Characterizing the Links Between Systemic Sclerosis and Breast Cancer

**DOI:** 10.7759/cureus.66653

**Published:** 2024-08-11

**Authors:** Aditya Dutt, Lauren Arcinas, Justin Chen, Rahila Shaikh, Sejoon Jun, Amritpal Kooner, Dhruv Gandhi, Conor Dolehide

**Affiliations:** 1 Dermatology, Midwestern University Chicago College of Osteopathic Medicine, Downers Grove, USA; 2 Dermatology, University of Ottawa, Ottawa, CAN; 3 Human Biology, Health, and Society, Cornell University, Ithaca, USA; 4 Dermatology, Walk-In Dermatology, Plainview, USA; 5 Medicine, K. J. Somaiya Medical College & Research Centre, Mumbai, IND

**Keywords:** antibodies, breast cancer, cancer, scleroderma, systemic sclerosis

## Abstract

Systemic sclerosis (SSc) is a complex, autoimmune connective tissue disease that affects multiple organs in the body, culminating in a variance of severity and a reduced quality of life. Breast cancer (BC) also affects patients with SSc, and these two conditions affect a similar demographic. With this systematic review, we aim to characterize the links between SSc and BC. Characterizing possible links between SSc and breast malignancies is important for advancing the understanding of SSc management and comorbidities. In this systematic meta-analysis, a comprehensive literature search was conducted in PubMed using relevant keywords and MeSH terms. The inclusion criteria included an English-language retrospective analysis that characterized patients with SSc with or without BC. Two independent reviewers assessed the study’s eligibility based on predetermined criteria. Data extraction included patient antibody measurements, demographics (age and gender), family history, social behaviors (alcohol use and smoking history), concurrent condition treatments, and adverse effects following treatment. Thirteen articles were identified in the literature with relevant data on SSc and BC patients. Studies encompassed research about SSc patients with or without BC and relevant risk factors being measured. SSc was found to have a link to antibodies widely associated with cancer. Adverse treatment outcomes and concurrent conditions of BC were found when patients had a family history of SSc, BC, or an alcohol or smoking history. Our results suggest that the presence of antinuclear antibodies, anti-centromere antibodies, or anti-topoisomerase antibodies in SSc patients is correlated with BC. Out of the three antibodies, ATA seemed to be found more commonly in patients with SSc and malignancy across the studies. This systematic review discusses the link between SSc and BC through patients with relevant clinical markers, medical histories, and treatments. However, further research is necessary to advance the linkage between SSc and BC and determine whether management of one condition may prevent or alleviate the other.

## Introduction and background

Systemic sclerosis (SSc), also known as scleroderma, is a multifaceted autoimmune connective tissue disorder that targets various organs, leading to a spectrum of clinical manifestations and a consequent decrease in life quality. Predominantly affecting women between the ages of 30-50, SSc presents a unique challenge not only in its complexity and variability but also in its management due to the breadth of organ involvement, including the GI, pulmonary, cardiac, and renal systems, among others [1]. This condition, characterized by the overproduction and accumulation of collagen in the tissues, leads to fibrosis, vascular abnormalities, and immune dysregulation [1].

Emerging evidence suggests a concerning link between SSc and an increased risk of malignancies, notably breast cancer (BC) [1]. BC, the most common cancer among women worldwide, shares an intriguing, yet not fully elucidated, association with SSc. The potential interplay between these two diseases raises important questions regarding the underlying mechanisms of autoimmune disorders and carcinogenesis. Particularly, the presence of specific antibodies commonly found in SSc patients, such as antinuclear antibodies (ANA), anti-topoisomerase antibodies (ATA), and anticentromere antibodies (ACA), has been associated with an increased risk of cancer, including BC [1]. These antibodies, indicative of an active autoimmune response, may play a role in the pathogenesis of malignancies or serve as biomarkers for identifying individuals at higher risk.

The impact of lifestyle and environmental factors, such as alcohol consumption and smoking history, on the risk of developing SSc and BC further complicates the relationship between these conditions [1]. Alcohol use and smoking have been independently linked to an increased risk of various cancers and autoimmune diseases, suggesting that these social behaviors could contribute to the observed association between SSc and BC.

Understanding the links between SSc and BC is crucial not only for elucidating the pathophysiological mechanisms underpinning these diseases but also for improving patient management and outcomes. By investigating these demographics, family history, social factors, and treatment efficacies in patients with both SSc and BC, this systematic meta-analysis aims to shed light on the complex interactions between these conditions. Characterizing the potential connections between SSc and breast malignancies is pivotal for advancing our understanding of SSc management, comorbidities, and the overarching implications for patient care and prognosis.

## Review

Methods

A systematic review was conducted using the Preferred Reporting Items for Systematic Reviews and Meta-Analyses (PRISMA) checklist. Four electronic databases - PubMed, Scopus, Embase, and Web of Science - were examined from inception to January 20, 2024. The identified articles were blind-screened by two independent researchers. All conflicts were resolved by an unbiased third researcher.

**Figure 1:**
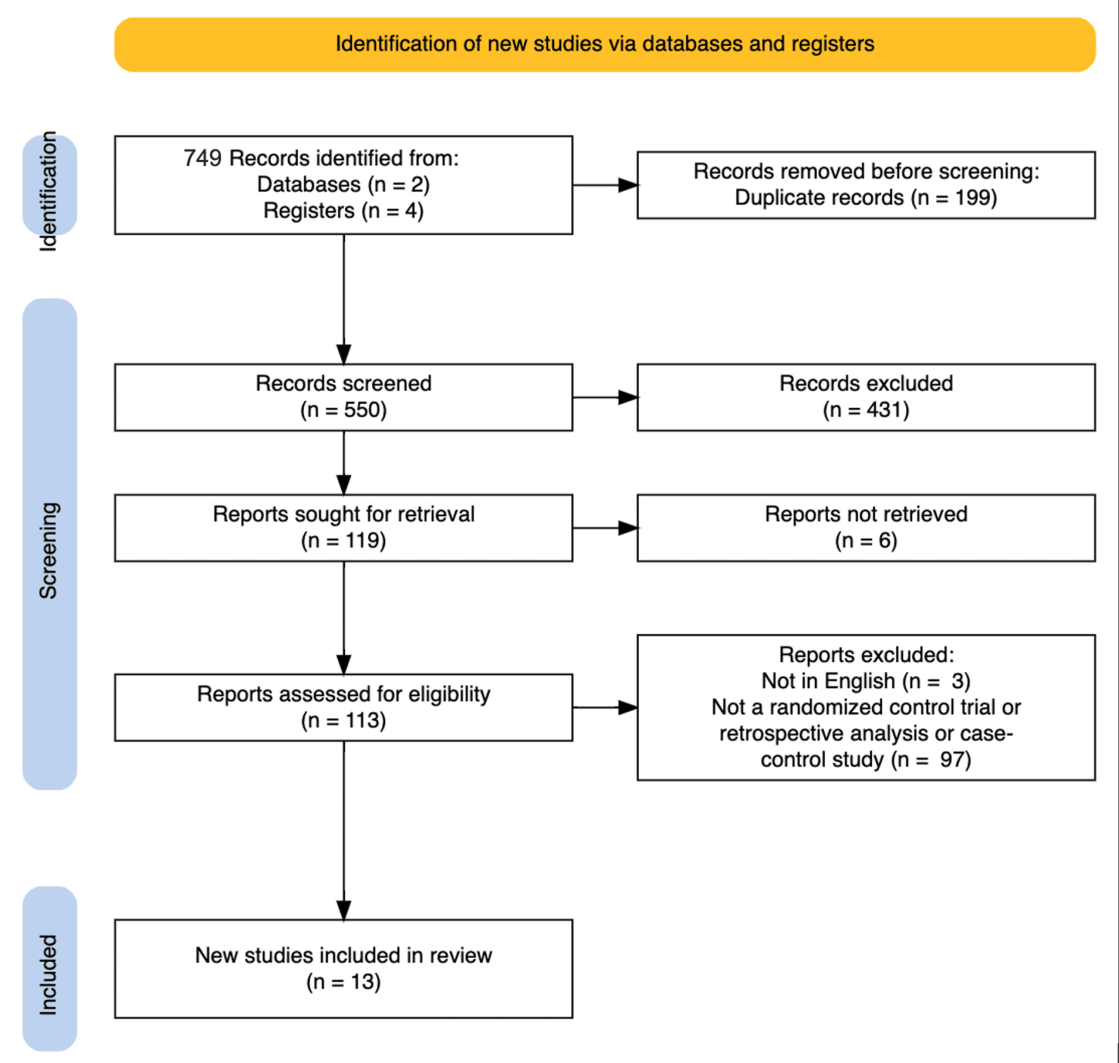
PRISMA flow diagram PRISMA, Preferred Reporting Items for Systematic Reviews and Meta-Analyses

**Figure 2:**
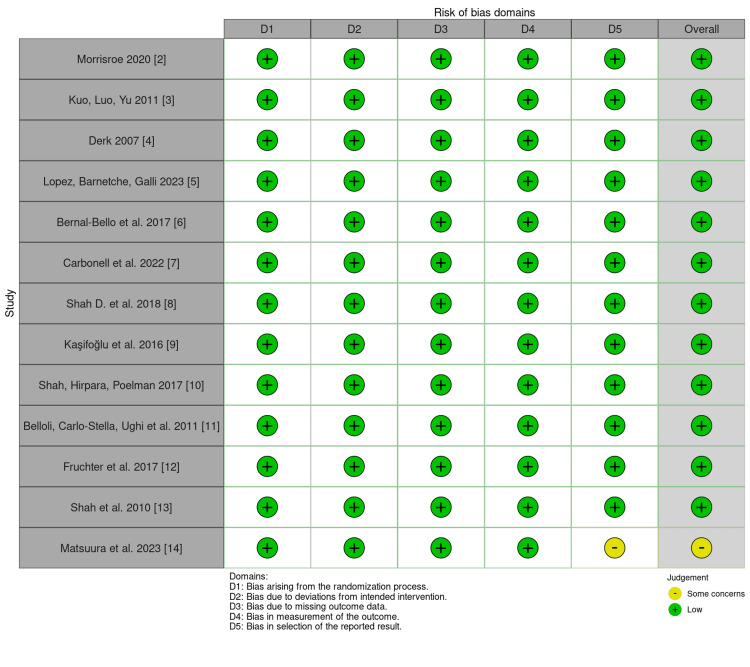
Risk of bias plot

The search was based on studies that discussed patients with scleroderma and BC. Article selection focused on selecting articles that contained relevant data on SSc and BC (or generalized cancer if BC was not delineated in cancer types) from randomized control trials, retrospective analyses, or case-control studies. A PRISMA flow chart was generated in Figure 1 based on the steps completed for the review. The remaining articles were then screened for a risk of bias assessment, as detailed in Figure 2.

Eligibility Criteria

The following criteria were included: (1) randomized control trials, retrospective analysis, and case-control studies; (2) reports of scleroderma and (3) BC; and (4) the investigation provided reliable data that could be examined, including the total number of participants and insightful outcomes of each metric.

The following criteria were excluded: (1) research that did not provide adequate information about experimental or control group results; (2) in vitro and animal studies; (3) research reported in foreign languages; (4) meta-analyses, systematic reviews, and other reviews (excluding primary sources); (5) studies lacking full-text or only presenting abstracts; and (6) non-randomized controlled trials, cohort studies, case reports, and case series.

A systematic literature review was conducted to assess the relationship between scleroderma and BC in terms of diagnosis, treatment, and patient outcomes. This review aimed to analyze the existing literature, identify any gaps, and gain insights into the current understanding of scleroderma and BC.

Results

Age

According to the analyzed studies with comparable SSc and generalized cancer average ages, the average age at SSc diagnosis was lower than the average age at generalized cancer diagnosis. In a 2020 study characterizing cancer in relation to SSc in the general Australian population, the average age at SSc onset was 46.6 years (SD = 14.3 years), while the average age of generalized cancer diagnosis was 57.7 years (SD = 12.9 years) [2]. A study characterizing the Taiwanese population reported similar trends, where the average age for male and female SSc onset was 51.6 (SD = 17.9) and 47.3 (SD = 15.3) years of age, respectively, and the average age for male and female generalized cancer diagnosis was 59.1 (SD = 13.3) and 58.0 (SD = 13.8) years of age, respectively [3].

In Derk’s study, compared to 48 randomly selected SSc patients without a cancer diagnosis, the SSc patients with BC were diagnosed with SSc at an older age (53.5 +/- 12.2) compared to those without (42.4 +/- 12.5) [4]. His study also showed that in pre-SSc BC patients (n = 13), the age at SSc and BC diagnosis was 61.6 (SD = 13.3) and 56.2 (SD = 10.2), respectively [4]. For patients diagnosed with SSc following BC (n = 11), the average age of SSc and BC diagnosis was 43.9 (SD = 13.5) and 51.2 (SD = 10.8) years of age, respectively [4]. For patients purely SSc positive with no BC, the mean age of SSc diagnosis was 42.4 years of age (SD = 12.5) [4].

Lopez et al.’s study found that SSc patients without malignancy were diagnosed with SSc at a mean age of 51 years of age (SD = 15.4), while SSc patients with malignancy were diagnosed with SSc at a mean age of 57 (SD = 13.8) [5].

A retrospective analysis of patients in a Spanish hospital presented data on SSc patients with and without diagnosed cancers. In patients without diagnosed malignancy, the age at SSc onset had been reported as 45.2 (SD = 16.2) years of age [6]. In patients with diagnosed malignancy, the age at SSc onset had been reported as 42.7 (SD = 15.9) years of age [6]. In a study analyzing Spanish Scleroderma registry data, patients without cancer at SSc onset were aged on average 45.9 (SD = 16.1) years, while patients with cancer at SSc onset were aged on average 49.3 years of age (SD = 17.0) [7].

In studies that had comparable SSc and BC average age data, the average age at SSc diagnosis was also lower than the average age at BC diagnosis [8]. A study examining patients from Johns Hopkins (JH) and the University of Pittsburgh (UoP) revealed that SSc patients who received no radiation therapy treatment, on average, had their SSc onset at 48.7 (SD = 14.4) and 51 (SD = 12.3) years of age from the two universities, respectively [8]. Those same SSc patients had also been diagnosed with BC at 56.8 (SD = 11.5) and 57.6 (SD = 11.5) years of age, from JH and UoP, respectively [8].

From the same study, SSc patients who had received radiation therapy treatment, on average, had their SSc onset at 50.4 (SD = 13.5) and 50.8 (SD = 12.5) years of age, from JH and UoP, respectively [8]. Those same patients who had received radiation therapy treatment, on average, had been diagnosed with BC at 53.7 (SD = 9.2) and 57.1 (SD = 9.7) years of age, respectively [8]. While the trend of SSc diagnosis preceding cancer diagnosis (in this case, BC) holds, it can also be noted that patients who received radiation therapy had later BC diagnoses than patients who did not receive radiation therapy when compared to the patients at the same sites [8].

Gender and Ethnicity

The intersection of SSc and BC primarily concerns the female population, given the prevalence of both conditions among women. This focus reflects the gendered nature of both diseases, with SSc occurring more frequently in women than men at a ratio that varies in literature but is significantly skewed towards females [2]. BC, being the most common cancer among women worldwide, similarly centers this demographic in studies exploring the link between SSc and BC.

The predominance of SSc and BC in women is a reflection of both biological susceptibility and sex-specific environmental exposures. Biologically, female women being disproportionately affected at ratios reported as high as 4:1 in some studies underscores the need for gender-specific research and clinical approaches [5]. Hormonal factors, including the roles of estrogen and progesterone, are believed to contribute to this disparity, influencing immune system function and the development of autoimmune conditions. Similarly, BC’s high incidence among women highlights the importance of gender in understanding cancer epidemiology, necessitating targeted screening and prevention strategies that address the unique hormonal and genetic profiles of female patients [5].

Moreover, the gender perspective is critical when considering patient experiences and outcomes. Women with SSc may face unique challenges, including issues related to fertility, pregnancy, and psychosocial stressors, which can compound the complexity of managing their condition alongside BC. These aspects underscore the importance of a holistic, gender-sensitive approach to care that addresses the full spectrum of patients’ needs and experiences.

The studies included in the meta-analysis encompass a diverse array of patient backgrounds, including populations from Australia, the United States, Spain, and Taiwan. This diversity allows for a broad examination of how SSc and BC intersect across different ethnic and cultural backgrounds. Genetic predispositions unique to certain ethnic groups can influence disease susceptibility, severity, and patient outcomes [2]. For instance, genetic variants associated with SSc and BC risk may have different frequencies across ethnic groups, leading to variations in disease incidence and progression patterns. Additionally, cultural factors and social determinants of health, including diet, lifestyle, and access to healthcare, can significantly impact disease outcomes.

Studies from diverse geographic regions, such as Australia, the United States, Spain, and Taiwan, contribute to a global understanding of SSc and BC. However, these studies also highlight the importance of contextualizing findings within specific ethnic and cultural frameworks. For example, the impact of environmental exposures, such as occupational hazards or pollution, may vary significantly between countries and cultures, influencing disease risk and outcomes [3].

In the Australian context, SSc and its association with cancer were examined in a comprehensive study that provided insight into how these conditions manifest within the Australian population [4]. Similarly, research conducted in the United States offered a glimpse into the intersection of these diseases among American patients, highlighting the need to consider how ethnic diversity within the country might impact the findings.

The Spanish studies added valuable data on European populations, contrasting findings from non-European contexts and enriching the understanding of SSc and BC in a Mediterranean demographic [7]. The inclusion of research from Taiwan brought an East Asian perspective, which is crucial for understanding how SSc and BC are present and managed in Asian populations [3].

Access to healthcare and health literacy levels also vary widely across different ethnic groups and geographic regions, affecting early detection, treatment options, and patient prognosis. Inequities in healthcare access can lead to delayed diagnoses and treatment, particularly for BC, where early detection is crucial for improving survival rates. Culturally competent care and targeted health education campaigns are essential for addressing these disparities, ensuring that all patients, regardless of ethnicity or location, receive timely and effective interventions.

This broad geographic and ethnic diversity within the study populations underscores the importance of considering a wide range of factors when examining the links between SSc and BC. It suggests that while some risk factors and disease characteristics may be universal, others may vary significantly across different populations. Understanding these nuances is critical for developing tailored approaches to the management and treatment of SSc and BC in diverse patient populations.

When examining the intersection of SSc and BC through the lens of sex and ethnicity, it is paramount to quantify the sex disparity and examine the ethnic-specific disease incidence and outcomes. For instance, Morrisroe et al. (2020) observed that among the Australian population, women represented approximately 75% of the SSc cases, highlighting a significant gender disparity inherent to the disease [2]. This aligns with global estimates, where SSc is known to affect women more frequently than men.

In terms of ethnicity, Kuo et al. (2012) reported on the Taiwanese population, noting differences in the onset age of SSc and associated cancer risks between different ethnic groups [3]. While the study primarily focused on the Taiwanese population, it suggested potential variances that could be influenced by genetic backgrounds [3]. The research indicates that ethnicity may play a critical role in disease susceptibility and progression, emphasizing the need for studies that specifically address these differences. For instance, the incidence rate of SSc and BC in Caucasian populations compared to Asian populations could provide valuable insights into how ethnic backgrounds influence disease dynamics [3].

Moreover, Bernal-Bello et al. (2017) and Carbonell et al. (2022) highlighted the necessity of considering ethnic and cultural contexts in understanding the prevalence and management of SSc and BC [6,7]. By examining the data across various populations, researchers can begin to outline the impact of ethnicity on disease outcomes, potentially identifying unique risk factors or protective elements inherent to specific ethnic groups.

Smoking and Alcohol 

The impact of smoking and alcohol consumption on the risk and progression of SSc and BC represents a crucial area of investigation within the broader context of lifestyle factors and their influence on autoimmune and malignant conditions. Both smoking and alcohol consumption have been independently linked to an increased risk of various cancers and autoimmune diseases, suggesting that these behaviors could play a significant role in the observed association between SSc and BC.

Smoking, a known risk factor for numerous health conditions, has been specifically implicated in the pathogenesis of autoimmune diseases, including SSc [9]. It may exacerbate the vascular damage and fibrotic processes characteristic of SSc, thereby affecting disease severity and progression. In the context of cancer, smoking is a well-established risk factor for many types of malignancies, including BC, where it may influence cancer risk through mechanisms related to hormonal regulation, DNA damage, and systemic inflammation.

Alcohol consumption, similarly, has been associated with an increased risk of BC, with evidence suggesting a dose-response relationship where higher levels of consumption are linked to greater risks [8]. The mechanisms through which alcohol may increase BC risk include hormonal effects, as alcohol consumption can lead to elevated levels of estrogen and other hormones associated with BC. Additionally, alcohol can contribute to oxidative stress and DNA damage, further elevating cancer risk.

In patients with SSc, the role of alcohol is less clear, but it may influence disease outcomes through its effects on the immune system and potential to exacerbate organ damage. Given the systemic nature of SSc and its impact on multiple organs, including the GI tract, liver, and kidneys, alcohol consumption could potentially worsen existing conditions or increase the risk of complications [10].

For the impact of smoking and alcohol consumption on SSc and BC risk and progression, the available data allows for a more specific trend analysis. According to Shah et al. (2018), patients with a history of smoking had a notably higher incidence of both SSc and BC compared to non-smokers [10]. For example, in their cohort, smokers constituted approximately 30% of the SSc patients, a figure significantly higher than the general population smoking rates, indicating a potential correlation between smoking and increased SSc risk [10].

In terms of alcohol consumption, the same study indicated that moderate to heavy drinkers (defined as consuming more than 14 alcoholic drinks per week) represented around 20% of the SSc patients, a percentage that again exceeds general population consumption patterns [10]. This suggests a possible link between alcohol consumption and the heightened risk of SSc, although the direct correlation to BC risk within this subgroup remains less clear, underscoring the need for further investigation.

The exploration of smoking and alcohol consumption in the context of SSc and BC highlights the importance of lifestyle factors in disease risk and progression. Understanding how these behaviors interact with the biological processes underlying SSc and BC is critical for developing comprehensive management strategies. It underscores the need for patient education and lifestyle modifications as part of a holistic approach to disease management, aiming to reduce risk factors and improve outcomes for patients with SSc, BC, or both.

Antibodies

Prior literature on SSc and BC has documented a similarity between the antibodies that these two conditions share, such as ACA, ANA, and anti-Scl-70 antibodies (also known as ATA) [1].

ACA 

In the study analyzing the Australian population, out of the 1,727 total SSc participants, 245 (14.2%) were found to have cancer, while 1,482 (85.8%) had no cancer. A total of 1,656 of the 1,727 SSc patients had positive ACA results [2]. The level of ACA among the SSc participants with cancer was 107/245 (44.9%), and without cancer, it was 689/1,482 (48.6%) [2]. The study never specified how many of those participants who tested positive for ACA had breast malignancy, specifically.

Kaşifoğlu et al.’s study had 340 SSc-diagnosed participants, 315 without malignancy, and 25 with malignancy [9]. Forty-four (13.9%) SSc patients without malignancy tested positive for ACA, while four (16%) of SSc patients with malignancy tested positive for ACA [9]. The study never specified how many participants with malignancies had BC or how many of those participants who tested positive for ACA had BC [9].

Lopez et al.’s study had 464 SSc patients, 74 (16%) of whom had cancer. Overall, 220 (47.4%) of the 464 patients tested positive for ACA [5]. A total of 188 (48.2%) out of 390 SSc patients without malignancy tested positive for ACA, while 32 (43%) of the 74 SSc patients with malignancy tested positive for ACA [5]. The study never specified how many participants with malignancy had BC or how many of those participants who tested positive for ACA had BC [5].

Derk’s study had a greater level of result stratification in terms of organizing participants with SSc and BC [4]. 24 participants had SSc and BC, with 6/24 (25%) testing positive for ACA. 48 participants had SSc without BC, with 13/48 (27.1%) testing positive for ACA [1]. One stratification included 13 participants who had BC that preceded a BC diagnosis, and it was found that four (30.1%) of them had tested positive for ACA [1]. The next stratification included 11 participants who had BC and followed the BC diagnosis, and it was found that 2/11 (18.2%) of participants tested positive for ACA [1]. Finally, out of 48 participants who had SSc without BC, 13/48 (27.1%) tested positive for ACA [1].

In Bernal-Bello et al.’s study (2017), there were 423 patients who had been tested for ACA [6]. Out of 53 SSC patients with malignancy, 21 (39.6%) had tested positive for ACA, compared to out of 370 SSC patients without malignancy, 155 (41.9%) tested positive for ACA [6].

In Belloli et al.’s 2011 study, 8/14 (57.1%) of SSc patients with cancer tested positive for ACA, while 56/98 (57.1%) of SSc patients without cancer tested positive for ACA [11]. The study never specified how many of those participants who tested positive for ACA had breast malignancy, specifically [11].

Shah et al.’s study that examined the temporal relationship between SSc and cancer onset had 23 individuals diagnosed with cancer, of whom 13 (56.5%) had BC specifically [8]. Of the 23 individuals, eight (34.8%) tested positive for ACA (denoted as CENP in this study) [8]. The study never specified how many of those participants who tested positive for ACA had breast malignancy, specifically [8].

In Carbonell et al.’s 2022 study, there were 1,935 SSc patients, 206 of whom had cancer as well [7]. The study did not have antibody concentrations consistently added, so the numbers did not add up completely. A total of 761/1545 (49.3%) SSc patients without cancer tested positive for ACA, while 73/179 (40.8%) SSc patients with cancer tested positive for ACA [7]. The study never specified how many participants with malignancy had BC or how many of those participants who tested positive for ACA had BC. The study did note that the presence of ACA lowered cancer risk [7].

Shah et al.’s study (2018) was split between JH and UoP SSc-cancer patients who had or had not received radiation treatment [8]. It should be noted that antibodies were not always consistently measured in patients [8]. Of the 43 SSc-cancer JH patients who did receive radiation, 12/41 (29.3%) tested positive for ACA [8]. Of the 73 SSc-cancer JH patients who did not receive radiation, 22/67 (32.9%) tested positive for ACA. Of the 26 SSc-cancer UoP patients who did receive radiation, 6/21 (28.6%) tested positive for ACA [8]. Of the 11 SSc-cancer UoP patients who did not receive it, none tested positive for ACA [8].

ANA 

In the study analyzing the Australian population, out of the 1,727 total SSc participants, 245 (14.2%) were found to have cancer, while 1,482 (85.8%) had no cancer [2]. A total of 1,101 out of the 1,727 total participants had positive ANA results [2]. The level of ANA among the participants with cancer was 23/245 (13.6%), and that without cancer was 132/1,482 (14.1%) [2]. The study never specified how many of those participants who tested positive for ANA had BC, specifically.

Interestingly, in the studies examined by us, we found higher rates of ANA positivity in patients with scleroderma without BC as compared to patients with scleroderma with BC [4]. Derk’s 2007 study found ANA positivity in 62.5% of patients with SSc who had BC, as compared to 89.5% of patients with SSc who had no association with BC [4]. This dearth of ANA positivity in the BC group was found to be statistically significant.

Kaşifoğlu et al.’s study analyzed 310 SSc patients without malignancy, and 25 SSc patients with malignancy [9]. They found that 282/315 (89.5%) of the SSc patients without malignancy tested positive for ANA, while 20/25 (80%) of the SSc patients with malignancy tested positive for ANA [9].

Lopez et al.’s 2023 study had 464 SSc patients; 74 (16%) had cancer and 390 without cancer. Out of the 390 SSc patients without cancer, 89/390 (22.8%) had tested positive for ANA [5]. Out of the 74 patients who did have cancer, 9 (12.2%) tested positive for ANA [5].

In Bernal-Bello et al.’s 2017 study, there were 432 SSc patients, with 53 having malignancies and 15 of those being breast malignancies [6]. A total of 417/431 (96.7%) of the SSc patients tested positive for ANA. Moreover, 367/378 (97.1%) of the patients with SSc only tested positive for ANA [6], while 21/53 (39.6%) of the patients with SSc and malignancy tested positive for ANA [6].

In Carbonell et al.’s 2022 study, there were 1,935 SSc patients, 206 of whom had cancer as well [7]. The study did not have antibody concentrations consistently added up, so the numbers did not add up completely [7]. A total of 39/376 (10.4%) recorded SSc patients without cancer were found to have tested positive for ANA [7]. Moreover, 10/42 (23.8%) of SSc patients with cancer were found to have tested positive for ANA [7].

Shah et al.’s study was split between JH and UoP SSc-cancer patients who had or had not received radiation treatment [8]. It should be noted that antibody concentrations are not always consistently measured in patients. A total of 9/31 (29%) recorded SSc patients from JH who had received radiation treatment had tested positive for ANA [8]. Moreover, 17/61 (27/9%) recorded SSc patients from JH who had not received radiation treatment tested positive for ANA [8], while 5/21 (23.8%) recorded UoP SSc patients who received radiation had tested positive for ANA. A total of 2/7 (28.6%) recorded UoP SSc patients who had not received radiation tested positive for ANA [8].

ATA

In the study analyzing the Australian population, out of the 1,727 total SSc participants, 245 (14.2%) were found to have cancer, while 1,482 (85.8%) had no cancer [2]. A total of 1,630 out of the 1,727 total participants had positive ATA results [2]. The level of ATA among the participants with cancer was 37/245 (15.7%), and the level without cancer was 212/1,482 (15.2%) [2]. The study never specified how many of those participants who tested positive for ATA had breast malignancy, specifically [2].

Kaşifoğlu et al.’s (2016) study had 340 SSc-diagnosed participants, 315 without malignancy, and 25 with malignancy [9]. A total of 145 (46%) SSc patients without malignancy tested positive for ATA, while 15 (60%) of SSc patients with malignancy tested positive for ATA [9]. The study never specified how many participants with malignancies had BC or how many of those participants who tested positive for ACA had BC [9].

Lopez et al.’s study had 464 SSc patients, of whom 74 (16%) had cancer [5]. Overall, 79 (17%) of the 464 patients tested positive for ATA [5]. A total of 76 (19%) out of 390 SSc patients without malignancy tested positive for ATA, while 19 (26%) of the 74 SSc patients with malignancy tested positive for ATA [5]. The study never specified how many participants with malignancy had BC or how many of those participants who tested positive for ACA had BC [5].

Derk’s 2007 study had a greater level of result stratification in terms of organizing participants with SSc and BC [4]. Twenty-four participants had SSc and BC, with 1/24 (4%) testing positive for ATA. Forty-eight participants had SSc without BC, with 5/48 (10%) testing positive for ATA [4]. One stratification included 13 participants who had BC that preceded a BC diagnosis, and it was found that none had tested positive for ATA [4]. The next stratification included 11 participants who had BC that followed BC diagnosis, where it was found that 1/11 (9%) of participants tested positive for ATA [4]. Finally, out of 48 participants who had SSc without BC, 5/48 (10.4%) tested positive for ATA [4].

In Bernal-Bello et al.’s study, there were 407 patients who had been tested for ATA [6]. Out of 51 recorded SSc patients with malignancy, nine (17%) had tested positive for ATA, compared to out of 356 recorded SSC patients without malignancy, 76 (21.3%) had tested positive for ATA [6].

In Belloli et al.’s 2011 study, 4/14 (28.6%) of SSc patients with cancer tested positive for ATA, while 10/98 (10.2%) SSc patients without cancer tested positive for ATA [11]. The study never specified how many of those participants who tested positive for ATA had breast malignancy, specifically [11].

Shah et al.’s study that examined the temporal relationship between SSc and cancer onset had 23 individuals diagnosed with cancer, of whom 13 (56.5%) had BC specifically [8]. Of the 23 individuals, five (21.7%) tested positive for ATA [8]. The study never specified how many of those participants who tested positive for ATA had breast malignancy, specifically [8].

In Carbonell et al.’s 2022 study, there were 1,935 SSc patients, 206 of whom had cancer as well [7]. The study did not have antibody concentrations consistently added, so the numbers did not add up completely. A total of 65/922 (7%) SSc patients without cancer tested positive for ATA, while 15/112 (13%) SSc patients with cancer tested positive for ATA [7]. The study never specified how many participants with malignancies had BC or how many of those participants who tested positive for ACA had BC. The study did note that the presence of ACA lowered cancer risk [7]. 

Shah et al.’s study was split between JH and UoP SSc-cancer patients who had or had not received radiation treatment [8]. It should be noted that antibodies were not always consistently measured in patients. Of the 43 SSc-cancer JH patients who did receive radiation, 8/40 (29.3%) tested positive for ATA [8]. Of the 73 SSc-cancer JH patients who did not receive radiation, 13/66 (19.7%) tested positive for ATA [8]. Of the 26 SSc-cancer UoP patients who did receive radiation, 5/21 (23.8%) tested positive for ATA [8]. Of the 11 SSc-cancer UoP patients that did not receive it, 2/7 (28.6%) tested positive for ACA [8].

Radiation Treatments

A review of the literature showed three articles that examined the possible association between SSc and radiation therapy status post-cancer diagnosis.

Lopez et al. delve into the clinical manifestations of cancer-associated SSc, unveiling two distinct phases in cancer development among SSc patients [5]. Among the 464 patients, 74 were diagnosed with cancer. Of these, 26 cases involved BC, and 13 cases involved lung cancer [5]. Among those with BC, four patients underwent chemoradiation therapy, while one patient with lung cancer received the same treatment [5]. The first phase implies a potential correlation between cancer and the autoimmune response in SSc individuals, as evidenced by the increased cancer incidence concurrent with the diagnosis of SSc [5]. This hints at a paraneoplastic phenomenon, where the immune system reacts to the presence of cancer cells. The second phase reveals another notable increase in cancer occurrence more than four years after SSc diagnosis [5]. This observation suggests that factors such as chronic inflammation, oxidative stress, fibrosis-related damage, and immunosuppressive treatments in SSc patients may create a conducive environment for carcinogenesis [5].

Fruchter et al. highlight 22 cases of postirradiation morphea (PIM), characterized by sclerotic plaques akin to idiopathic morphea [12]. Among these patients, 21 were diagnosed with BC. The majority developed SSc within five years post-irradiation [12]. The study hypothesized that the increased risk of BC may stem from the close proximity of the radiation target (breast tissue) to the skin, in contrast to other malignancies [12]. Treatment primarily involved topical or intralesional therapy, showing limited spontaneous regression [12]. 

Belloli et al. examined six cancer patients (nonspecified) who received chemoradiotherapy [11]. However, none of these individuals exhibited SSc characteristics consistent with paraneoplastic syndrome [11].

No definitive association between radiation therapy and systemic scleroderma has been observed. These articles underscore the need for continued research to unravel the relationship between systemic scleroderma and radiation therapy post-cancer diagnosis. To shed light on potential connections and the underlying mechanisms, future research endeavors should focus on more targeted approaches, emphasizing the necessity of larger sample sizes and specific study methodologies.

Non-Radiation Treatments

Nine articles discussed the use of non-radiation treatments, such as immunosuppressive therapy and chemotherapy. Of the immunosuppressive agents, the most commonly utilized include corticosteroids, methotrexate, azathioprine, and cyclophosphamide [4-6,9,12,13]. Corticosteroid use among SSc patients with malignancy was 29.23%, compared to 4.22% among SSc patients without malignancy [6,13]. Methotrexate was utilized among 13.33% of SSc patients with malignancy and 19.64% of SSc patients without malignancy [6,5,13]. The use of azathioprine among SSc patients with malignancy was 4.70%, compared to 7.02% among SSc patients without malignancy [4,5,12]. Cyclophosphamide use among SSc patients with malignancy was 40.09%, compared to 10.96% among SSc patients without malignancy [6,9,13].

Additional immune suppressors utilized by SSc patients included mycophenolic acid, hydroxychloroquine, tocilizumab, TNF inhibitors, and D-penicillamine. Derk (2007) reported that nine patients with BC (37.5%) were treated for SSc with D-penicillamine compared to 41.6% of SSc patients without BC, and three patients with BC (12.5%) were treated for SSc with methotrexate or cyclophosphamide compared to 12.5% of SSc patients without BC [4]. This study reported no statistically significant difference in immunosuppressive treatment use [4].

Morrisroe et al. (2020) reported treatments utilized by SSc patients with and without breast malignancy (n = 74 and n = 1,482, respectively) [2]. The extended period was defined as two consecutive years or more of therapy or a follow-up time greater than or equal to 50% in the Australian Scleroderma Cohort Study if the follow-up time was less than two years [2]. The association between an extended period of immunosuppression therapy and an increased risk of BC was not statistically significant (OR 0.79 (0.45-1.41), p = 0.430) [2]. Early-onset cancer was defined as being diagnosed within five years of SSc onset; results for SSc and early BC were also not statistically significant (OR 0.81 (0.29-2.27), p = 0.691) [2]. An extended period on calcium channel blockers was found to have a narrowly missed statistically significant correlation with an increased occurrence of breast malignancy (OR 1.61 (0.99-2.63), p = 0.051) [2]. However, the extended use of calcium channel blockers among patients with SSc was found to be associated with the occurrence of overall malignancy (OR 1.46 (1.06-2.02), p = 0.016) [2].

One article investigates the relationship between SSC and cancer, both malignant and non-malignant. It reveals significant findings regarding clinical characteristics, immunological markers, and risk factors.

Lopez et al. (2023) conducted a thorough investigation involving 464 SSc (SSc) patients, with 74 individuals diagnosed with cancer [5]. Their analysis unveiled significant associations between cancer and SSc, revealing that cancer-diagnosed patients tended to be older at the time of SSc diagnosis and experienced a higher mortality rate [5]. Furthermore, the presence of GI damage and nonspecific interstitial pneumonia was notably more common in SSc patients with concurrent cancer [5]. Immunological markers also emerged as crucial indicators. SSc patients demonstrated a higher frequency of positivity for anti-RNA Pol 3 and anti-SSA antibodies compared to those without malignancies [5]. A logistic regression analysis highlights the importance of individual risk factors, emphasizing the presence of these antibodies alongside the timing of SSc diagnosis and duration since cancer diagnosis. The study also investigated the timing of cancer onset concerning the SSc diagnosis, with a mean duration of approximately 3.5 years between the two diagnoses [5].

Additionally, the article studied various treatment options used for SSc patients, both with and without malignancy [5]. Disease-modifying agents, including hydroxychloroquine, methotrexate, azathioprine, mycophenolic acid, cyclophosphamide, rituximab, and tocilizumab, were administered at varying frequencies among the patient groups [5]. Symptomatic treatments such as proton-pump inhibitors, calcium channel blockers, endothelin receptor antagonists, phosphodiesterase-5 inhibitors, prostaglandin analogs, and acetylsalicylic acid were also utilized [5]. Notably, rituximab was used more commonly in SSc patients with synchronous cancer, while hydroxychloroquine was favored in cases with a history of cancer [5].

Overall, the study explores the potential relationship between SSc and cancer, providing valuable insights into clinical characteristics, immunological markers, risk factors, and treatment patterns among SSc patients. Further research and personalized approaches to optimize patient care should be sought.

Patient Outcomes

Morrisroe et al. (2020) explored SSc patients with cancer, revealing not only higher mortality rates but also increased hospitalizations and healthcare expenditures, with an annual excess per patient cost of A$1,496 (p < 0.001) [2]. The study detailed the impact of cancer on quality of life, highlighting the need for comprehensive supportive care interventions [2].

Kuo et al. (2012) analyzed 2,053 (472 men and 1,581 women) Taiwanese individuals diagnosed with SSc during 1996-2008 and 83 (30 men and 53 women) had cancer [3]. The incidence of cancer was 6.9/1,000 person-years [3]. The most common cancer site in male SSc patients was the lung (n = 10), and in females, it was the breast (n = 11) [3]. Compared to the Taiwanese population of 1996, the all-cancer SIR for SSc was 1.63 (95% CI 1.31-2.01) [3]. A cancer diagnosis in SSc patients was associated with a hazard ratio of 2.15 (95% CI 1.30-3.53). Among cancer patients, a diagnosis of SSc was not associated with increased mortality [3]. This study investigated long-term survival outcomes and cancer recurrence rates in SSc patients, providing valuable prognostic information for clinicians and patients alike [3]. Their findings underscored the importance of tailored treatment strategies and ongoing surveillance [3].

Lopez et al. (2023) studied the influence of cancer diagnosis timing on SSc disease progression and mortality rates [5]. This study examined 464 SSc patients; 74 (16%) had cancer, with BC (n = 26) being one of the most frequent [5]. The diagnosis of cancer was made less than three years before or after the SSc diagnosis for 23 patients (31%) [5]. In a multivariate analysis, anti-RNA Pol 3 and anti-SSA antibodies were significantly associated with an increased overall risk of cancer with an OR of 4.12 (95% CI (1.6-10.7); p < 0.01) and 2.43 (95% CI [1.1-5.4]; p < 0.05), respectively [5]. Age at diagnosis of SSc and delay from the SSc diagnosis were also independent risk factors for cancer [5]. Interstitial lung disease and ATA were associated with an increased risk of lung cancer and cancer occurring more than three years after SSc diagnosis [5]. In addition to anti-RNA Pol 3 antibodies, anti-SSA antibodies are associated with an increased risk of cancer in SSc patients [5]. Early cancer detection significantly improved survival and disease management outcomes in SSc patients [5].

Derk (2007) analyzed treatment response rates and disease-free survival outcomes in BC patients with coexisting SSc, highlighting the challenges of managing comorbid conditions [4]. Their study emphasized the importance of multidisciplinary care teams and personalized treatment approaches [4]. Compared to 48 randomly selected SSc patients without a diagnosis of cancer, the patients with BC were diagnosed with SSc at an older age (53.5 +/- 15.2) as compared to those without (42.4 +/- 12.5) (p = 0.002) [4]. A relatively equal amount of patients had the diffuse and limited form of SSc, while pulmonary fibrosis (p = 0.015) and the lack of ANA positivity (p = 0.02) were more commonly seen in patients with BC [4]. Patients who developed BC before the diagnosis of SSc were older at SSc diagnosis (61.6 +/- 11.3) compared to those after (43.9 +/- 13.5) (p = 0.03) [4]. An older age at diagnosis of SSc, a lack of ANA positivity, and the presence of pulmonary fibrosis were more commonly seen in patients with SSc who have a diagnosis of BC [4].

Shah et al. (2018) investigated radiation therapy-related complications and treatment adherence rates in BC patients with concurrent SSc, elucidating factors influencing treatment tolerance and adherence [8]. They analyzed 43 of the 116 BC patients at JH and 26 of the 37 patients at the UoP who received breast radiation therapy [8]. At JH, four of 30 (13.3%) patients with available data developed erythema, none had blistering, one of 30 (3.3%) developed ulceration, and 15 of 31 (48.4%) had skin thickening in the radiation port [8]. At the UoP, seven of 11 patients (63.6%) with available data developed erythema, two of 11 (18.2%) had blistering, none developed ulceration, and six of 11 (54.6%) had skin thickening in the radiation port [8]. In a limited sample, there were no significant changes in the mRSS or FVC% between patients who did and those who did not receive radiation therapy [8]. Their study provided valuable insights into optimizing radiation therapy protocols for this complex patient population.

Shah et al. (2010) explored the association between specific scleroderma autoantibodies and cancer recurrence rates, providing valuable insights into disease monitoring and surveillance strategies [13]. They evaluated 23 patients, with six testing positive for anti-RNA polymerase I/III, five for anti-topoisomerase I, eight for anticentromere, and four not positive for any of these antigens [13]. The median duration of scleroderma at cancer diagnosis differed significantly between groups (-1.2 years in the anti-RNA polymerase I/III group, +13.4 years in the anti-topoisomerase I group, +11.1 years in the anticentromere group, and +2.3 years in the group that was negative for all antigens tested) (p = 0.027) [13]. RNA polymerase III demonstrated a robust nucleolar staining pattern in 4 of 5 available tumors from patients with antibodies to RNA polymerase I/III [13]. In contrast, nucleolar RNA polymerase III staining was not detected in any of the four examined tumors from the RNA polymerase antibody-negative group (p = 0.048) [13]. Their results suggested that scleroderma autoantibodies are cancer biomarkers only in patients with clinical manifestations of specific rheumatic diseases and are unlikely to improve risk stratification for cancer in the general population [13]. Their findings informed discussions regarding personalized risk stratification and targeted intervention approaches.

Matsuura et al. (2023) explored the impact of SSc on BC treatment outcomes, revealing potential implications for disease recurrence and long-term survival [14]. They studied 25 patients with BC and collagen disorder between 2004 and 2011 [14]. The mean age was 56.4 ± 12.6 years, and 14, eight, and three patients had cancer of clinical stages I, II, and III, respectively, and only one patient had scleroderma as a comorbid collagen disorder. The expression statuses of hormone receptors (HR) and human epidermal growth factor receptor 2 (HER2) were HR(+), HER2(+), and HR(‑)HER2(‑) in 20 (80.0%), four (16.0%), and four (16.0%) patients, respectively. A total of 22 (84.0%) patients received steroids or immunosuppressive drugs for collagen disorders [14]. The collagen disorder group had a higher mean Ki-67 labeling index than the control group (41.1 vs. 20.8%; p = 0.007) [14]. After median observation periods of 103 and 114 months, the relapse-free survival (RFS) and overall survival (OS) rates were lower in the collagen group than in the control group (64.5 and 80.7% vs. 85.3 and 94.3%, respectively; p < 0.01) [14]. Patients with BC and collagen disorders had relatively high Ki‑67 expression and relatively low RFS and OS rates [14]. Their findings informed discussions regarding treatment selection and postoperative management strategies.

Bernal-Bello et al. (2017) analyzed malignancies diagnosed in 53 patients (12.2%). Fifty-eight neoplasms were identified, among which breast (n = 15) was one of the most prevalent [6]. In 19 patients, the diagnosis of both scleroderma and tumors was made <3 years apart [6]. Cancer significantly decreased the probability of survival (OR = 2.61; 95%CI 1.46-4.69; p = 0.001). The risk of cancer was directly associated with the presence of anti-PM/Scl antibodies (OR = 3.90; 95%CI 1.31-11.61; p = 0.014), which remained independent risk factors for cancer on multivariate analysis [6]. They reported that the presence of anti-PM/Scl antibodies remained independent risk factors for cancer on multivariate analysis, underscoring their potential role as prognostic indicators in SSc patients.

Fruchter et al. (2017) investigated long-term treatment response rates and disease recurrence outcomes in BC patients developing PIM [12]. There were 21 cases of PIM in BC, including one case of parotid gland carcinoma [12]. All 22 patients developed PIM within the irradiated site, and 50% of patients had disease extending beyond the treatment area [12]. Patients treated with phototherapy or systemic medications were more likely to exhibit marked clinical responses compared to those receiving only topical or intralesional therapy (89% vs. 22% with follow-up data; p = 0.015) [12]. Although over half of our PIM cohort was treated with topical or intralesional therapy alone, patients treated with systemic agents, most often methotrexate or narrowband ultraviolet-B phototherapy, were significantly more likely to achieve a markedly complete response [12]. Their study provided valuable insights into the efficacy of different treatment modalities and informed discussions regarding optimal therapeutic approaches for this rare complication.

Belloli et al. (2011) analyzed an Italian cohort of SSc patients to examine whether clinical and/or laboratory SSc-specific features represent a risk for developing malignancies in these patients [11]. Fifteen cancers were found in 14 patients, and the majority (60%) occurred after SSc onset (average 16 years), and 40% occurred before the onset of SSc (average 14 years) [11]. The most frequent was BC (prevalence: 4.5%, relative prevalence: 33.3%). Cancers were unrelated to SSc type, autoantibodies, organ involvement, and treatments [11]. Their study revealed that cancers were unrelated to SSc type, autoantibodies, organ involvement, and treatments.

Carbonell et al. (2022) evaluated 1930 patients with SSc; 206 of these patients had cancer, with BC being one of the most common [7]. Patients with SSc had increased risks of overall cancer (SIR 1.48, 95% CI 1.36-1.60; p < 0.001) and BC (SIR 1.31, 95% CI 1.10-1.54; p = 0.003) [7]. Cancer was associated with older age at SSc onset (OR 1.22, 95% CI 1.01-1.03; p < 0.001), the presence of primary biliary cholangitis (OR 2.35, 95% CI 1.18-4.68; p = 0.015) and forced vital capacity <70% (OR 1.8, 95% CI 1.24-2.70; p = 0.002) [7]. The presence of ACA lowered the risk of cancer (OR 0.66, 95% CI 0.45-0.97; p = 0.036) [7]. 

Kaşifoğlu et al. (2016) evaluated 340 SSc patient medical records for malignancy [9]. Twenty-five SSc patients had 19 different types of malignancies, with bladder cancer being the most common and BC being the second [9]. There were 282 patients found to have antibodies in both groups [9]. Of the patients without malignancy, 282 had ANA antibodies (89.5%), 145 had Scl-70 (ATA) (46%), and 44 had ACA (13.9%) [9]. In comparison to the patients with malignancy, 20 had ANA (80%), 15 had Scl-70 (ATA) (60%), and four had ACA (16%) [9]. 

Shah et al. (2010) evaluated 23 participants with SSc and cancer diagnoses, and 13 out of the 23 patients had BC [13]. The average age at SSc diagnosis was 50.1 +/- 12.1, and 22 of the 23 participants were female [13]. 22 of the 23 participants were also White, and one was Black [13]. Six patients tested positive for anti-RNA polymerase I/III, five for anti-topoisomerase I, eight for anticentromere, and four were not positive for any of these antigens [13]. The median duration of scleroderma at cancer diagnosis differed significantly between groups, -1.2 years in the anti-RNA polymerase I/III group, +13.4 years in the anti-topoisomerase I group, +11.1 years in the anticentromere group, and +2.3 years in the group that was negative for all antigens tested (p = 0.027) [13]. RNA polymerase III demonstrated a robust nucleolar staining pattern in 4 of 5 available tumors from patients with antibodies to RNA polymerase I/III [13]. In contrast, nucleolar RNA polymerase III staining was not detected in any of the four examined tumors from the RNA polymerase antibody-negative group (p = 0.048) [13]. The study concluded a close temporal relationship between the onset of cancer and scleroderma in patients with antibodies to RNA polymerase I/III, which is distinct from scleroderma patients with other autoantibody specificities. In this study, autoantibody response and tumor antigen expression are associated [13].

Adverse Effects

Fruchter et al. (2017) documented post-irradiation morphea as an adverse outcome following radiation treatment, although other potential adverse effects of radiation were not addressed [12]. Among the other studies reviewed, no adverse effects were reported concerning the treatments administered [12].

Discussion

The results gleaned from the age section of demographics across all the studies with relevant data supported the general trend that the average age at SSc diagnosis was before the average age at generalized cancer diagnosis. In Morrisroe et al.’s 2020 study characterizing the Australian population findings, SSc onset on average preceded a cancer diagnosis by around 11 years [2]. Similar findings were echoed in Ku et al.’s 2012 study, which found that SSc diagnosis would precede generalized cancer diagnosis by around a decade for both males and females [3].

Patients with only SSc had been diagnosed at an earlier age than patients with SSc and a cancer diagnosis. Lopez et al.’s 2023 study supported this, with SSc-only patients being diagnosed with SSc on average around six years earlier than patients with SSc and malignancy [5]. This may support the notion that genotypes or factors that promote malignancy may also promote SSc. In Bernal-Bello et al.’s (2017) study, it was found that patients with only SSc had reported their onset of SSc at an age around three years before the age of SSc onset that patients with SSc and a cancer diagnosis had reported [6]. A similar finding was also found in Carbonell et al.’s (2022) study, with SSc patients without cancer having an onset of SSc on average at an age of around three years prior to SSc onset in patients with SSc and cancer [7]. It is important to note that some studies depended on patients reporting an onset, rather than a diagnosis, of SSc.

Derk’s 2007 study looked at SSc and BC specifically [4]. Results demonstrated that patients diagnosed with BC, then SSc, were diagnosed at older ages with these conditions than patients with SSc, then BC, or patients diagnosed only with SSc [1]. Patients with only an SSc diagnosis were diagnosed on average at a younger age than patients with an SSc diagnosis either following or preceding BC.

Shah et al.’s 2018 study analyzing JH and UoP patients also found that SSc diagnosis on average preceded BC diagnosis [8]. Notably, their study also analyzed radiation treatment, which seemed to find that SSc and BC patients who had received radiation therapy had been diagnosed with BC at a younger age, as opposed to SSc and BC patients who had not received radiation therapy [8]. It should, however, be considered that the degree of BC in the participants was not measured, so perhaps the type of BC that the older patients with SSc had did not make them a good candidate for radiation treatment [8].

With the exception of Shah et al.’s 2010 study, ACA positivity was found to be more evident in patients with solely SSc in comparison to SSc and a diagnosed malignancy. In other words, those with SSc and malignancy seemed to test negative for ACA on average more often than patients with just SSc. Studies whose results supported this included those done by Morrisroe et al. (2020), Derk (2007), Kaşifoğlu et al. (2016), Lopez et al. (2023), Bernal-Bello et al. (2017), and Carbonell et al. (2022) [2,4-7,9].

With the exception of Carbonell et al.’s 2022 study, ANA positivity was found to be more evident in patients with solely SSc in comparison to SSc and a diagnosed malignancy. In other words, those with SSc and malignancy seemed to test negative for ANA on average more often than patients with just SSc. Studies whose results supported this included those done by Morrisroe et al. (2020), Derk (2007), Kaşifoğlu et al. (2016), Lopez et al. (2023), and Bernal-Bello et al. (2017) [2,4-6,9].

ATA positivity analysis in the studies yielded interesting results, with some studies documenting ATA positivity being higher in patients with both SSc and malignancy. Studies whose results revealed that ATA positivity was higher in patients with both SSc and a malignancy included those done by Morrisroe et al. (2020), Kaşifoğlu et al. (2016), Belloli et al. (2011), and Carbonell et al. (2022) [2,7,9,11]. Studies whose results detracted from this trend included Derk (2007) and Bernal-Bello et al. (2017) [4,6].

Taking the findings across the studies as a whole, it could be accurate to say that ANA and ACA positivity may be an indicator for solely SSc, however, ATA positivity may be an indicator for SSc and a malignancy diagnosis. These findings suggest that the positivity of ACA, ANA, and ATA could be useful in screening and treating SSc patients with or without malignancy.

Lopez et al. (2023), Belloli et al. (2011), and Fruchter et al. (2017) explore the potential relationship between SSc and radiation therapy following a cancer diagnosis; however, a conclusive association has not been established [5,11,12]. The majority of BC patients in Fruchter et al.’s 2017 study developed SSc within five years following radiation treatment. None of the cancer patients in Belloli et al.’s 2011 study who received chemoradiotherapy developed SSc characteristics consistent with paraneoplastic syndrome [11]. Additional research is required to elucidate whether a definitive association exists between radiation therapy and SSc.

Limitations

The limitations of Morrisroe et al.’s 2020 study included smaller patient numbers in some cancer types, limiting the incidence of the SSc cancer cohort and decreasing the reliability of determining the factors associated with cancer in this specific cohort [2]. Additionally, the high-resolution CT (HRCT) was only performed in patients with a clinical suspicion of interstitial lung disease or abnormal RFT, which may cause selection bias and overdiagnosis of lung cancers in those who underwent an HRCT compared with those who did not undergo an HRCT [2].

In Derk’s 2007 study, the limitations were based on the study’s design due to the sample population being obtained from a subspecialty clinic at a university-based hospital, carrying some selection bias [4]. The sample population was also smaller, and certain differences between groups were clear; however, a larger sample size would allow for other differences that were not of statistical significance to be better analyzed [4]. Furthermore, there may be a bias in the study due to the pulmonary fibrosis prevalence in non-BC controls being lower than previously described in scleroderma patients [4]. 

The limitations of Kaşifoğlu et al. (2016) included the retrospective design of the study [9]. Evaluating certain differences was difficult due to this study design, such as the inability to evaluate the severity of organ involvement. Additionally, the RNA polymerase III antibody was not evaluated routinely. Matsuura et al. (2023) also performed a retrospective study with clinicopathological factors obtained from hospital medical records [14]. The sample size in this study of both intervention and control groups was overall small. The Fruchter et al. (2017) study was also influenced by the retrospective design of the study and the possibility of spontaneous resolution of the post-irradiation morphea [12]. Bernal-Bello (2017) et al. also performed a retrospective study, with a small number of patients included from a single-referral center, and certain antibody samples, such as anti-RNA pol-III, were not performed in all patients [6].

Kuo et al.’s 2012 study included multiple limitations. Firstly, sample population classification was based on SSc and cancer diagnosis as recorded in the catastrophic illness registry [3]. This registry required a comprehensive investigation by the physician-in-charge and the Bureau of National Health Insurance (NHI) [3]. Patients with mild severity of disease may not have been registered and therefore not included in this study [3]. Additionally, other clinical characteristics such as SSc type (localized, limited, or diffuse) and laboratory parameters were not recorded in the NHI database [3]. Secondly, SSc patients are more likely to seek medical attention compared to the general population, leading to ascertainment bias [3].

The limitations of Lopez et al.’s 2023 study include that the type of anti-SSA antibodies was not identified, and not all patients were screened for anti-RNA Pol III (ANA) [5]. Mixed connective tissue disease patients who tested positive for anti-RNP and with the SSc phenotype were included, adding more heterogeneity to the cohort, and these patients tested more often negative for other specific autoantibodies (ANA, ATA, and ACA) [5].

Shah et al.’s 2017 study limitations include a small sample size [10]. This also did not include additional BC patients because at least 2% of the patients exhibited moderate-strong autoantibody positivity [10]. A majority of the BC patients had a first- or second-degree relative with a history of BC; however, 18% of patients were sporadic [10]. Overall, this study population may limit the generalizability of the study’s findings.

Belloli et al.’s 2011 study was unable to evaluate certain related factors, such as family history, or risk factors, such as smoking or pregnancy [11]. Shah et al.’s 2010 study was limited by the retrospective design, small sample size, and ability to generalize the conclusions of the study [10]. This study also lacked serum samples during malignancy onset and changes in autoantibody profiles in response to cancer treatment [10]. The Carbonell et al. 2022 study’s limitation included sample sizes available for certain neoplasias, and data on risk factors for specific cancers was not collected [7].

Shah et al.’s 2018 study limitations include the inability to examine the type of radiation therapy, the extent of the radiation field, and dose-related effects on the patients [8]. Side effects from radiation, such as telangiectasias, were not reported, and a retrospective chart review was used to determine cutaneous and pulmonary outcomes rather than the Radiation Therapy Oncology Group scoring criteria for acute and late toxicities [8]. The severity of radiation-induced skin thickening was not determined due to the retrospective review [8]. The data was also obtained from tertiary care centers and may overestimate radiation-induced fibrosis risk in scleroderma patients with BC [8]. Overall, there was a lack of control of the population without scleroderma in this study [8].

It also should be noted that we did not fully look at the relationship between scleroderma and BC in the context of examining autoantibody epitopes in patients with BC. There may be a potential relationship between the autoantibody epitopes of SSc patients and whether the BC in these patients may be related to the epitopes on their autoantibodies.

## Conclusions

SSc is a rare autoimmune disease that is multifaceted in nature, targeting various organs and resulting in a variety of symptoms and a reduced quality of life. Determining the relationship between SSc and malignancies, particularly BC, is crucial for improving patient management and outcomes. This systematic review reports that SSc diagnosis typically precedes BC diagnosis by a decade; patients diagnosed solely with SSc are generally younger than those who develop both SSc and cancer. The data suggests that specific autoantibodies can be valuable markers in screening and managing SSc patients. ACA and ACA positivity appears more prevalent in patients with only SSc, whereas ATA positivity is higher in those with concurrent malignancies. This highlights the potential for these autoantibodies to aid in distinguishing between SSc patients with and without cancer, potentially improving screening accuracy and patient care.

Current findings suggest patients diagnosed with BC following an SSC diagnosis are younger compared to those diagnosed with SSc after developing BC. However, the relationship between SSc and radiation therapy remains inconclusive, warranting additional research. As such, several limitations were noted, and addressing these limitations in future research through larger cohorts with a control group is essential. Overall, while this systematic review provides valuable insights into the relationship between SSc, cancer diagnoses, and the potential use of autoantibodies as markers, further research is necessary to fully understand the underlying mechanisms.
